# Uterine artery Doppler and endometrial blood flow in frozen embryo transfer: A cohort study

**DOI:** 10.18502/ijrm.v21i3.13196

**Published:** 2023-04-14

**Authors:** Fereshteh Bahrami, Maryam Eftekhar, Leila Zanbagh

**Affiliations:** ^1^Research and Clinical Center for Infertility, Yazd Reproductive Sciences Institute, Shahid Sadoughi University of Medical Sciences, Yazd, Iran.; ^2^Department of Obstetrics and Gynecology, Afshar Hospital, Shahid Sadoughi University of Medical Sciences, Yazd, Iran.

**Keywords:** Doppler ultrasonography, Embryo transfer, Assisted reproductive technology, Pregnancy.

## Abstract

**Background:**

The implantation rate after assisted reproductive technology depends on 2 important factors, good quality embryo and endometrial receptivity. Endometrial receptivity is mainly assessed by ultrasound measurement of endometrial thickness and morphology.

**Objective:**

This study aimed to investigate the relationship between uterine artery Doppler indices/endometrial perfusion and pregnancy rate.

**Materials and Methods:**

This cohort study was done on 250 women who were candidates for frozen embryo transfer from January to July 2022. For assessing endometrial receptivity, we performed a Doppler ultrasound of the uterus before embryo transfer with apparently desirable endometrium (endometrial thickness ≥ 7 mm and 3 line endometrial pattern). In addition, the women were divided into 2 groups according to assisted reproductive technology outcome (clinical pregnancy), group I positive clinical pregnancy, and group II negative clinical pregnancy, and uterine artery indices and endometrial perfusion were compared between these groups.

**Results:**

Uterine artery Doppler showed that the pulsatility index was significantly different between positive and negative clinical pregnancy groups, but resistance index and peak systolic velocity (PSV) did not have statistically significant differences. Also, endometrial perfusion was significantly different between the 2 groups of clinical pregnancy. Endometrial perfusion was significantly better in positive clinical pregnancy groups.

**Conclusion:**

Doppler ultrasound can help to assess endometrial receptivity.

## 1. Introduction

One of the most important steps in assisted reproductive technology (ART) is proper embryo transfer. Embryo implantation and successful pregnancy require a good quality embryo, a receptive endometrium, and their coordination (1). Endometrial receptivity depends on its thickness and morphological changes, which are influenced by hormonal changes during menstrual cycles or exogenous hormone administration. It also appears that sufficient vascular circulation affects endometrial receptivity (2).

Numerous methods have been suggested in the literature to assess endometrial receptivity. These methods include measuring the endometrial thickness (ET), evaluating endometrial pattern, endometrial receptive analysis (ERA), estimating endometrial perfusion, and uterine artery Doppler (3, 4). An endometrial receptive analysis is a costly and invasive method. Ultrasound is valuable for inspecting receptivity before embryo transfer. If ultrasound Doppler, along with ET and morphology can be utilized to analyze the endometrial receptivity, this can be introduced as a cost-effective and noninvasive method.

Several studies have been conducted on the effectiveness of endometrial perfusion and uterine artery Doppler as indicators for endometrial blood flow. They have investigated the relationship between uterine/endometrial perfusion and implantation. Decrease in endometrial perfusion and abnormal uterine artery velocity have been mentioned as related factors in unexplained infertility (5-8). However, whether these factors affect pregnancy rate in in-vitro fertilization (IVF) cycles remains unclear. Karishme et al. (9) have found Doppler as an effective indicator for implantation rate, these results are not confirmed by another study (10).

Uterine artery flow indices may not indicate proper endometrial perfusion. It may be due to the collateral ovarian artery and the effect of myometrium on endometrial perfusion (11).

Here, we aim to investigate the impact of endometrial perfusion and uterine artery Doppler in frozen embryo transfer (FET) cycles.

## 2. Materials and Methods 

In this cohort study 250 women candidates for FET, aged 18-40 yr, referred to Research and Clinical Center for Infertility, Yazd, Iran from January to July 2022 were studied. All women with severe male factor (azoospermia), egg donation, and uterine surrogacy were excluded.

Demographic characteristics including age, anti-Mullerian hormone level, body mass index, and the cause and duration of infertility were extracted from the participants record, and data collection form were filled out for each participant individually.

**Figure 1:**
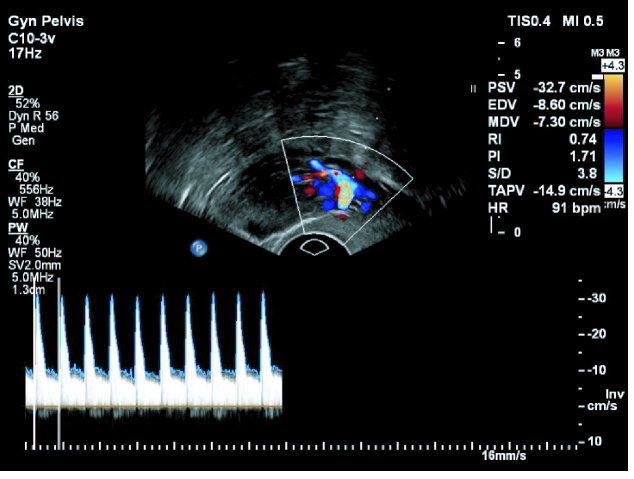
Uterine artery Doppler, by color Doppler ultrasound.

**Figure 2:**
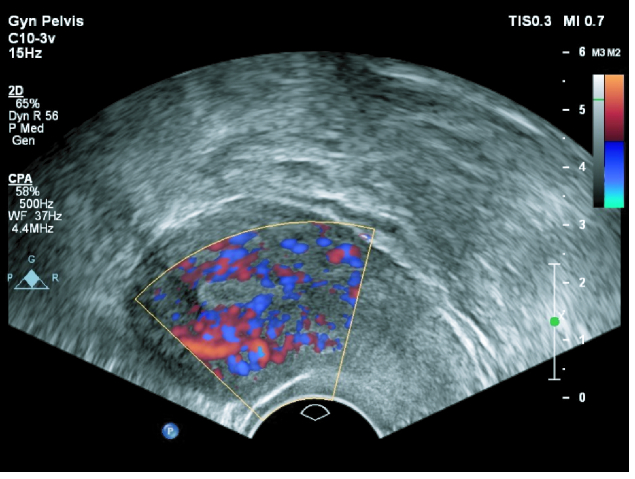
Endometrial perfusion by power Doppler ultrasound.

All participants had received routine FET cycle protocol. Ultrasound was done for all women on the 2 ${}^{\text{nd}}$ day of the menstrual cycle. When there was a quiescent ovary, hormone replacement therapy was started with estradiol 6 mg/day until days 12-13. In case of ET ≥ 7 mm and 3-line pattern, a Doppler ultrasound was done by a single gynecologist on a sagittal view. When the uterus and cervix were seen in one view, uterine artery blood flow was measured in ascending branch at the level of cervical internal os. We used pulse wave Doppler and set the gate on 2/3 vessel diameter and angle of insinuation on 60%. Assessed uterine artery indices were pulsatility index (PI = PSV-end-diastolic velocity/mean velocity), resistance index (RI = PSV- end-diastolic velocity/PSV), and peak systolic velocity (PSV). Measurement of endometrial blood supply was done by power Doppler (Figure 1). According to the presence of spiral blood vessels in 3 regions of the endometrium zone 1) endomyometrial junction, 5 mm of sub-endometrial area, zone 2) hyper-echo endometrial edge, zone 3) internal endometrial hypo-echo area (Figure 2); both Doppler sonographies were done on a sagittal view of the uterus by a 7.5 MHZ vaginal probe of a Philips affinity 70 machine.

Embryo transfer was done in the cleavage stage after 3 days of progesterone suppositories 400 mg twice daily plus 50 mg progesterone (intra muscular daily injection) (Aburaihan Pharmaceutical Company, Iran).

Participants were divided into 2 groups based on the results of clinical pregnancy (positive [group I] and negative [group II] clinical pregnancy) and followed-up 2-3 wk after a positive pregnancy test (beta human chorionic gonadotropin ≥ 50). Finally, the mean of uterine artery blood flow indices and zone of spiral artery supply were compared between groups.

### Sample size

Sample size were determined considering the confidence level of 95% and power of tests 80%, based on the standard deviation of 1.12 for uterine artery flow variable and the mean difference of 0.3. The sample size was calculated as 250 samples using G.power software (version 3.1).

### Ethical considerations

The study protocol was approved by the Ethics Committee of the Yazd Reproductive Sciences Institute, Shahid Sadoughi University of Medical Sciences, Yazd, Iran (Code: IR.SSU.RSI.REC.1399.046). Written informed consent was obtained from all participants before study.

### Statistical analysis

Statistical analysis was done using SPSS software (Statistical Package for the Social Sciences, version 18, Chicago, IL, USA). Cycle characteristics were compared between 2 groups using the Mann-Whitney and Kruskal-Wallis for continuous variables and the Chi-square test for categorical variables. P-value < 0.05 was considered as significant.

## 3. Results 

Initially, 374 women candidates for FET, aged 18-40 yr, were included in this study. Due to exclusion criteria 61 women were excluded for severe male factors, 47 women were excluded for egg donation, 9 for uterine surrogacy, and 7 for blastocyst transfer. Finally, ultrasound Doppler study and statistical analysis was done for 250 women.

Among these 250 women, the clinical pregnancy rate was 29.6%. Basic and cycle characteristics, including age, AMH, BMI, ET, duration of infertility, quality of the transferred embryo, and cause of infertility, were comparable in both groups (positive and negative clinical pregnancy (Table I).

There was no statistical difference between group I and II in RI and PSV of uterine artery Doppler, but PI of uterine artery was significantly higher in the nonpregnant group (p = 0.03) (Table II).

Regarding endometrial perfusion, there was a significant difference in the zone of perfusion between pregnant and nonpregnant women, and 64 from 74 pregnant women had endometrial perfusion in zone 2 and 3 with p < 0.001 (Table III). Zone 1 endometrial blood flow increased the risk of negative clinical pregnancy rate (4 times) compared to zone 2 (relative risk, RR, 4.09; 95% confidence interval, CI, 1.91-8.78). In comparison to zone 3, zone 1 increased 13.5 times the risk of negative pregnancy rate (relative risk, RR, 13.5; 95% confidence interval, CI, 5.46-33.32).

Women were classified into 2 age groups ( < 35 [n = 186] and ≥ 35 [n = 64]) in order to assess the endometrial perfusion; however, endometrial perfusion was found to be comparable between these 2 subgroups (p = 0.18). In younger age groups 34.4%, 46.8%, 18.8% were in zone I, II, and III endometrial perfusion respectively. In age ≥ 35, there were 45.3%, 43.85, and 10.9% in zone I, II and III respectively.

Also, we assessed the correlation between PI of the uterine artery and endometrial perfusion, there was a decremented pattern of PI value from zone 1 to zone 3, but the difference was not statistically different, p = 0.15 (Table IV).

**Table 1:** Demographic characteristics of the participant in the study groups


**Variables**	**Group I (n = 74)**	**Group II (n = 176)**	**P-value**
Age (yr) *	31.2 ± 5.06 MD: 31.0/ IQR: 8.0	31.72 ± 5.16 MD: 32.0/ IQR: 7.0	0.37
AMH (ng/ml) *	5.74 ± 4.15 MD: 4.85/ IQR: 5.35	4.73 ± 3.25 MD: 4.1/ IQR: 4.5	0.12
BMI (kg/m^2^) *	26.18 ± 3.87 MD: 26.0/ IQR: 6.0	26.10 ± 3.45 MD: 26.0/ IQR: 4.0	0.50
Endometrial thickness (mm) *	8.77 ± 1.69 MD: 8.4/ IQR: 1.95	8.88 ± 1.41 MD: 8.5/ IQR: 2.0	0.78
Duration of infertility (yr) *		7.04 ± 3.78 MD: 7/ IQR: 5.25	6.99 ± 3.42 MD: 6/ IQR: 5.75	0.99
Good quality embryo**		73/74 (98.6)	164/176 (93.2)	0.08
Cause of infertility**				
	**PCOs**	35 (47.3)	72 (40.9)	
	**Male**	12 (16.2)	30 (17)	
	**Unexplained**	13 (17.6)	39 (22.1)	
	**Mix**	4 (5.4)	14 (8)	
	**Tubal**	2 (2.7)	1 (0.6)	
	**Diminished ovarian reserve**	6 (8.1)	17 (9.7)	
	**Endometriosis**	2 (2.7)	2 (1.1)	
	**Hypothalamic amenorrhea**	0 (0)	1 (0.6)	0.68
*Data presented as Mean ± SD, Mann-Whitney. **Data presented as n (%), Chi-square, AMH: Anti-Mullerian hormone, BMI: Body mass index, MD: Median, IQR: Interquartile range, PCOS: Polycystic ovary syndrome				

**Table 2:** Comparison of uterine artery Doppler in 2 study groups


**Uterine artery Doppler indices**	**Group I (n = 74)**	**Group II (n = 176)**	**P-value**
**RI**	0.77 ± 0.22 MD: O.83/ IQR: O.22	0.82 ± 0.19 MD: O.85/ IQR: 0.19	0.24
**PI**	2.0 ± 0.82 MD: 1.9/ IQR: 1.23	2.31 ± 0.99 MD: 2.10/ IQR: 1.23	0.03
**PSV**	36.62 ± 6.97 MD: 38/ IQR: 5	35.90 ± 6.29 MD: 38/ IQR: 5	0.49
Data are presented as Mean ± SD, Mann-Whitney test, RI: Resistance index, PI: Pulsatility index, PSV: Peak systolic velocity, MD: Median, IQR: Interquartile range			

**Table 3:** Comparison of zone of endometrial perfusion in 2 study group


**Endometrial grade**	**Group I (n = 74)**	**Group II (n = 176)**	**P-value**
**Zone 1 (n = 93)**	10/74 (13.5)	83/176 (47.2)	
**Zone 2 (n = 115)**	38/74 (51.4)	77/176 (43.8)	
**Zone 3 (n = 42)**	26/74 (35.1)	16/176 (9.1)	< 0.001
Data presented as n (%). Chi-square test			

**Table 4:** Correlation of uterine artery PI and endometrial perfusion


**Endometrial grade**	**PI**	**P-value**
**Zone 1**	2.32 ± 1.01 MD: 2.20/ IQR: 1.12	
**Zone 2**	2.21 ± 0.87 MD: 2.10/ IQR: 1.40	
**Zone 3**	1.93 ± 0.96 MD: 1.80/ IQR: 0.79	0.15
Data presented as Mean ± SD. Kruskal-Wallis test. PI: Pulsatility index, MD: Median, IQR: Interquartile range		

## 4. Discussion

In this study, we investigated the impact of endometrial blood flow and uterine artery Doppler indices on the pregnancy rate of FET cycles. We found that the PI of the uterine artery in the positive clinical pregnancy group was significantly lower than in negative clinical pregnancy. In zone 3 of endometrial perfusion, 61% (26/42) of women achieved pregnancy, whereas in zone 1, 89% (83/93) cases did not. Therefore, it seems that there may be a correlation between endometrial perfusion and endometrial receptivity.

Also, we estimated the relative risk of negative pregnancy associated with endometrial blood flow, which revealed that zone 1 increases negative pregnancy rate in comparison to zone 2 and 3 (4 and 13.5 times respectively). These clear differences may suggest that endometrial blood flow assessment can be consider in routine clinical evaluation to improve pregnancy rate in FET cycle, in combination with ET.

It is well-known that during the menstrual cycle, the endometrial artery resistance reduces from the follicular to mid-luteal phase, and this reduction in resistance continues in the case of pregnancy. However, such changes during ART cycles are not evident. Medications, existing pathologies in the reproductive system of infertile people, and the supraphysiologic estrogen in plasma hurt vasculature of endometrium. Hence, endometrial and sub-endometrial blood flow appear lower in ART than in natural cycles (2).

To investigate the impact of uterine, especially the endometrial blood supply in infertile women, several studies have used uterine artery Doppler and evaluations of endometrial and sub-endometrial blood supply among those with unexplained infertility. These studies have reported reduced endometrial blood flow and increased uterine artery resistance index among the group with unexplained infertility compared to the control group (5, 7, 8). Moreover, one study has linked the existence of sub-endometrial blood flow in intra uterine insemination cycles with increased pregnancy rates (11).

Several studies have focused on the link between uterine artery Doppler and implantation, often contradicting observations (9, 12-16). In one study on 200 infertile women, uterine artery Doppler and perfusion of endometrium were done. In this research, the pregnancy rate was higher with ET of 7-14 mm, 3-line endometrium, and favorable Doppler study (lower PI in pregnant group and zone 3 endometrial perfusion) (12). Another study on 65 women, assessed uterine artery Doppler, RI, and PI, which were lower and PSV was higher in the pregnant group, on the day of human chorionic gonadotropin injection (13).

On the other hand, several studies reported no association between pregnancy rate and uterine artery Doppler indices (9, 14, 15). Uterine artery Doppler on 60 women was evaluated and no significant difference in uterine artery Doppler indices between pregnant and nonpregnant women were reported. The limitation of this study was the small sample size (9). Another researcher experimented on 188 women during the ART cycles and found that uterine artery Doppler indices were not anticipating pregnancy rates in ART cycles. This study was done on fresh embryo transfer cycles (14). In another article on 106 women, RI and PI were lower, and PSV was higher in the pregnant group, but the differences were not statistically significant (15).

Several researchers demonstrated the effect of uterine artery Doppler indices and endometrial perfusion together (1, 17). A study conducted on 100 women found that pregnancy is a remote possibility with ET < 8 mm, no 3-lines in appearance, and unfavorable endometria blood supply. However, there was no correlation between pregnancy rate and uterine artery Doppler (17). Another study on 236 cases of candidates for IVF reported the association of pregnancy rate with endometrial blood flow. However, they did not find a correlation between pregnancy rate and uterine artery indices (1).

Several articles just assessed endometrial perfusion and found that pregnancy rate correlated with the degree of endometrial perfusion (6, 10, 18, 19). For instance, in a study which was done on 82 women in an ART cycle, endometrial volume and vascularity were higher in pregnant cases compared to nonpregnant cases (10). Another study on 165 persons found that the presence of endometrial blood flow improves IVF outcomes (4). Also correlation between endometrial perfusion and pregnancy rate was showed in another study on 101 women (6). However, 2 studies did not show that correlation, the first one in 2014 on 34 cases (18) and another in 2018 on 62 cases (19), both of which had a small sample size. The contradicting results reported in the literature appear to have been caused by differences in cycles characteristics, stimulation protocols, type of the transfer cycle, and the day of the cycle in which the ultrasound was performed. As mentioned, a more comprehensive study that analyzes the impacts of uterine artery Doppler and endometrial perfusion on different causes of infertility is needed. In this study, a proper comparison was not possible in some infertility groups due to the low number of cases.

## 5. Conclusion

Endometrial perfusion in zones II and III improves the pregnancy rate. It appears that the combined measurements of uterine artery PI, endometrial perfusion, and ET can be good contributing factors for pregnancy rate. Furthermore, our findings align with the generally accepted notion that a good endometrial blood supply is required for successful implantation.

##  Conflict of Interest

The authors declare that there is no conflict of interest.
